# Functionalised Oximes: Emergent Precursors for Carbon-, Nitrogen- and Oxygen-Centred Radicals

**DOI:** 10.3390/molecules21010063

**Published:** 2016-01-07

**Authors:** John C. Walton

**Affiliations:** EaStCHEM School of Chemistry, University of St. Andrews, St. Andrews, Fife KY16 9ST, UK; jcw@st-andrews.ac.uk; Tel.: +44-01334-463-864; Fax: +44-01334-463-808

**Keywords:** oxime esters, oxime ethers, free radicals, organic synthesis, photochemical reactions, EPR spectroscopy, photoredox catalysis, *N*-heterocycles

## Abstract

Oxime derivatives are easily made, are non-hazardous and have long shelf lives. They contain weak N–O bonds that undergo homolytic scission, on appropriate thermal or photochemical stimulus, to initially release a pair of *N*- and *O*-centred radicals. This article reviews the use of these precursors for studying the structures, reactions and kinetics of the released radicals. Two classes have been exploited for radical generation; one comprises carbonyl oximes, principally oxime esters and amides, and the second comprises oxime ethers. Both classes release an iminyl radical together with an equal amount of a second oxygen-centred radical. The *O*-centred radicals derived from carbonyl oximes decarboxylate giving access to a variety of carbon-centred and nitrogen-centred species. Methods developed for homolytically dissociating the oxime derivatives include UV irradiation, conventional thermal and microwave heating. Photoredox catalytic methods succeed well with specially functionalised oximes and this aspect is also reviewed. Attention is also drawn to the key contributions made by EPR spectroscopy, aided by DFT computations, in elucidating the structures and dynamics of the transient intermediates.

## 1. Introduction

**Scheme 1:**
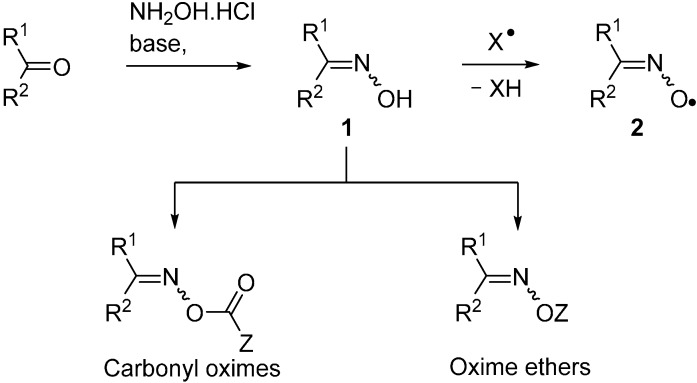
Oximes, carbonyl oximes and oxime ethers; production of iminoxyl radicals.

A huge variety of compounds containing the carbonyl functional group is available from natural and commercial sources. Of these, aldehydes and ketones can readily and efficiently be converted to oximes R^1^R^2^C=NOH (**1**) by treatment with hydroxylamine hydrochloride and a base. Alternatively, oximes can be prepared from reaction of organic and inorganic nitrites with various compounds containing acidic C–H atoms. Consequently, oximes are accessible in great diversity for further functional group transformations. A few instances of oximes themselves being used directly for radical generation are known. However, their O–H bonds are comparatively weak, usually in the range of 76–85 kcal·mol^−1^ [1] and, therefore, any radicals X^•^ generated in their presence abstract these H-atoms with production of iminoxyl radicals **2** (see Scheme 1). Many iminoxyls have been generated and studied [2,3,4] and they resemble nitroxide (aminoxyl) radicals in a number of ways; especially in that most are persistent. Frequently, therefore, the presence of an oxime serves to impede further radical reactions.

Oximes are easily derivatised so that oxime esters (*O*-alkanoyl and *O*-aroyl oximes) and oxime ethers (*O*-alkyl and *O*-aryl oximes) are straightforward to make. The N–O bonds in these compounds are usually comparatively weak; ~50 kcal·mol^−1^ in oximes [5] and only 33–37 kcal·mol^−1^ in *O*-phenyl oxime ethers [6]. Homolytic scission of these N–O bonds can be accomplished either by photochemical or by thermal means thus yielding for each type an *N*-centred radical accompanied by one equivalent of its *O*-centred counterpart. For this reason, oxime derivatives, especially carbonyl-oximes containing the >C=N–OC(=O)– unit, are finding increasing use as selective sources for free radicals. They offer tangible advantages over traditional initiators, such as diacyl peroxides, azo-compounds or organotin hydrides; all of which have well-known troublesome features. Most oxime derivatives are easily handled; are non-toxic, non-pyrophoric and have long shelf lives. The field has expanded to encompass a huge range of structural elements resulting in diverse and varied possibilities for subsequent transformations of the radicals. These compound types are being subsumed into more environmentally friendly preparative methods and for new means of access to ranges of aza-heterocycles. This article reviews modern methods of releasing radicals from both these precursor types. It highlights how their use has enabled the structures, reactions and kinetics of sets of *C*-, *N*- and *O*-centred radicals to be elucidated in greater detail than heretofore.

In earlier synthesis-orientated research, Forrester and co-workers employed oximeacetic acids MeCR(CH_2_)_2_CR^1^=NOCH_2_CO_2_H and their *t*-butyl peresters [7,8]. Hasebe’s group developed photochemical alkylations and arylations with oxime esters Ph_2_C=NOC(O)R [9,10] and Zard established ingenious preparative methodology from oxime benzoates PhRC=NOC(O)Ph [11], from *O*-benzoyl hydroxamic acid derivatives and from sulfenylimines R_2_C=NSAr [12,13,14]. Since the turn of the century, research has zeroed in on two particular sets of oxime-derived compounds: (a) carbonyl oxime derivatives and (b) *O*-aryl oxime ethers R^1^R^2^C=NOAr. Recent advances, important insights and details of some surprising outcomes are described with particular emphasis on mechanistic and computational aspects.

## 2. Oxime Esters and Related Carbonyl Oximes

### 2.1. General Features of Carbonyl Oxime Reactions

**Chart 1:**
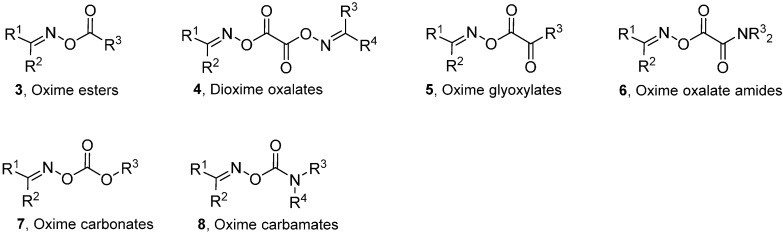
Types of carbonyl oximes investigated for radical release.

Generalised structures of the principal types of carbonyl oximes so far investigated are displayed in Chart 1. These carbonyl oxime types all have strong absorption bands in the 250–350 nm range and readily dissociate on UV irradiation in solution by cleavage of their N–O bonds. Cleavage of the O–C(O) bonds does not compete in solution under the normal conditions for organic reactions. Occasional observations of iminoxyl radicals (R^1^R^2^C=NO^•^), and products therefrom can most probably be attributed to residual trace impurities of the oximes from which the carbonyl oximes were prepared. Density Functional Theory (DFT) computations [B3LYP/6-31+G(d)] with model oxime esters **3** also indicated N–O cleavage was thermodynamically favoured over O–C(O) cleavage. It is apparent from Chart 1 that, by appropriate choice amongst these carbonyl oxime precursors, selective generation of radicals centred on either C-, N-, or O-atoms, with differing substitution patterns, can be achieved. The associated chemistry has been investigated spectroscopically and, of course, by end product analyses. The most successful and helpful method for mechanistic and dynamic information about the intermediates has been EPR spectroscopy coupled with QM computations. The synergy achievable by their mutual deployment has been demonstrated many times.

The efficiency of photochemical N–O bond homolysis depends strongly on the type of imine unit. Best radical yields were obtained when R^1^ and/or R^2^ were aromatic/heteroaromatic groups. Furthermore, incorporation of methoxy substituents in the aryl rings was usually also beneficial. In most photolyses, use of a photosensitizer such as 4-methoxyacetophenone (MAP) also proved advantageous. Energy transfer between the MAP excited state and the iminyl portion of the oxime derivative was probably promoted by π–π-stacking of their aryl units.

For all six types of carbonyl oxime (**3**–**8**), UV irradiation in hydrocarbon solutions, either straight or MAP sensitised, initially generated an iminyl radical (R^1^R^2^C=N^•^; designated **Im**) accompanied by an equivalent of a second species. All were therefore effective iminyl radical sources. Symmetrical dioxime oxalates **4** (R^1^ = R^3^ and R^2^ = R^4^) gave only one radical type and were particularly convenient for the study of iminyls. The discovery of these precursors permitted detailed EPR spectroscopic study of diverse iminyls’ structures and reactions. The second *O*-centred radicals released by carbonyl oximes (other than **4**) included acyls and carbamoyls as well as novel and exotic species such as alkoxycarbonyloxyl (alkyl carbonate radicals) R^3^OC(O)O^•^ and carbamoyloxyl R^3^R^4^NC(O)O^•^ radicals. Subsequent transformations enabled *C*-centred alkyl, acyl and carbamoyl radicals, as well as *N*-centred aminyl radicals, to be benignly generated. Much inaccessible information about the behaviour of these species therefore became reachable.

None of the carbonyl oxime precursors dissociate cleanly by thermolyses in the usual temperature range of organic preparations (*T* < ~120 °C). Flash Vacuum Pyrolysis (*ca.* 650 °C) led to electrocyclic rather than radical reactions though these also had useful preparative connotations [15].

### 2.2. Iminyl Radical Structures and Transformations

In the past, for spectroscopic purposes, specialised methods of generating iminyl radicals were employed. For example, iminyls were obtained from organic nitriles by electron bombardment and subsequent protonation and/or by H-atom addition [16]. Thermal dissociations of thionocarbamates [17], H-atom abstractions from imines [18] and treatments of organic azides with *t*-BuO^•^ radicals [19,20] were also put to use in obtaining iminyl EPR and other spectra. The range of accessible iminyl types has been considerably broadened as oxime derivatives **3**–**8** have come into use.

**Figure 1:**
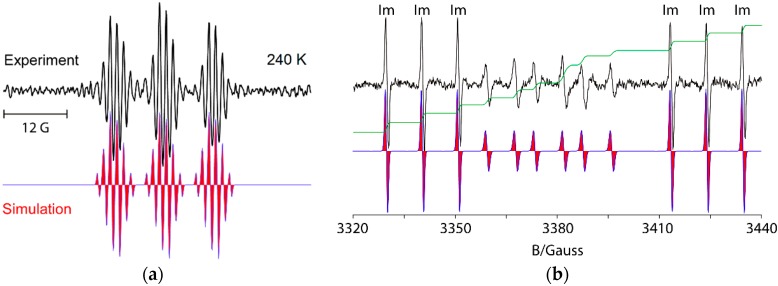
9 GHz isotropic EPR spectra of selected iminyl radicals in *t*-butylbenzene solution. **Left top** (**a**) Experimental spectrum of 1-phenylpropan-2-yliminyl radical **9**, with simulation **below** (**red**), during UV photolysis of dioxime oxalate **4a**; **Right top** (**b**) Experimental spectrum during UV photolysis of oxime ester **3a** at 320 K showing iminyl radical **11** (marked **Im**) in black with simulation in red (**below**) and double integral in green. The spectrum of adduct oxyaminyl radical **12** appears in the central region.

**Scheme 2:**
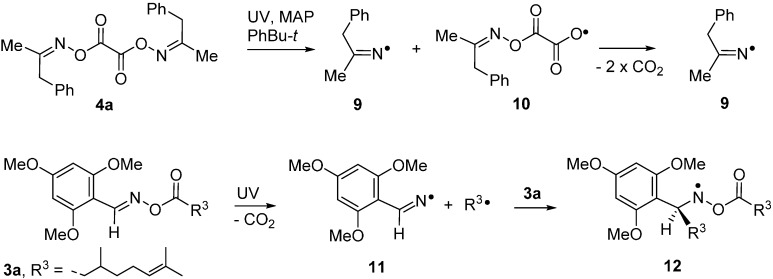
Photolytic generation of iminyl radicals from carbonyl oximes.

For example, UV irradiation of dioxime oxalate **4a** in *t*-butylbenzene solvent with MAP as photosensitizer, at 240 K, in the resonant cavity of a 9 GHz EPR spectrometer enabled the spectrum shown in Figure 1a to be obtained. Absorption of radiation led to scission of one of the N–O bonds and production of iminyl radical **9** plus *O*-centred radical **10**. The latter was probably extremely short-lived and dissociated with release of a second copy of **9** (Scheme 2). Thus, the EPR spectrum consisted of only iminyl **9** in the accessible temperature range. The 1:1:1 triplet hyperfine splitting (hfs) from the ^14^*N*-atom was slightly asymmetrical due to incomplete averaging of the radical tumbling at 240 K. Small hfs from the benzyl CH_2_ and the Me group were also resolved (Table 1).

The spectrum in Figure 1b illustrates the advantages of precursors prepared from aldoximes such as **3a**. The released ald-iminyls have structures R^1^CH=N^•^ with β-H-atoms. Because of the large hfs from its β-H-atom, the spectrum of the 2,4,6-trimethoxyphenyliminyl radical **11** appears as sharp 1:1:1 triplets in either wing (**Im**) leaving an extensive central “window” in the field where spectra of other radicals can appear well resolved. Ald-iminyl radical spectra act as valuable references for determination of the *g*-factors of other species. However, more importantly, photo-dissociation of **3a**, (and other carbonyl oximes) releases equimolar quantities of the iminyl and its partner radical. As Figure 1 shows, double integrations of such ald-iminyl spectra are usually easy and, hence, these iminyls also act as key references for the absolute and/or relative concentrations of other radicals generated. In the case of dissociation of oxime ester **3a**, the *O*-centred partner radical rapidly lost CO_2_ with production of primary *C*-centred radical R^3^^•^ (Scheme 1). At lower temperatures (~240 K), the spectrum of R^3•^ was observed, whereas at higher temperatures R^3•^ added to the precursor oxime ester and generated the *N*-centred radical **12**. The spectrum of oxyaminyl **12** was well resolved in the central window (Figure 1b) [21,22].

**Figure 2:**
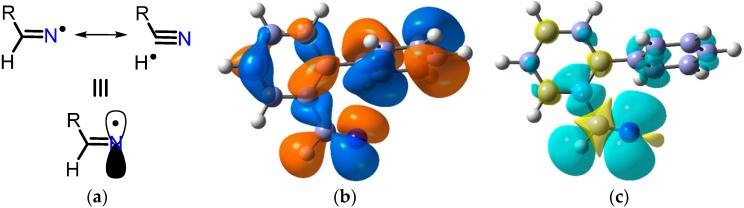
Electronic configuration of alkyl- and aryl-iminyl radicals. (**a**) hyperconjugative interaction; (**b**) DFT computed alpha SOMO; (**c**) DFT computed spin density distribution.

The EPR parameters of a representative set of iminyl radicals are collected in Table 1. They all have characteristic *g*-factors close to 2.0030 and isotropic *a*(^14^N) hfs of about 10 G. The magnitude of the latter is similar to that of π-type *N*-centred radicals indicating that the unpaired electron (upe) is mainly located in a nitrogen *2p* orbital [18]. In addition, the large values obtained for *a*(H^β^) point to a substantial hyperconjugative interaction such that the semi-occupied molecular orbital (SOMO) lies *in the nodal plane* of the C=N π-bond (Figure 2a).

**Table 1:** EPR Characteristics of iminyl radicals in solution ^a^.

Radical	Solvent	T/K	*g*-Factor	*a*(^14^N)	*a*(H^β^)	*a*(Other)	Reference
H_2_C=N^•^	*c*-C_3_H_6_	223	2.0028	9.7	85.2(2H)	-	[19]
MeHC=N^•^	H_2_O	300	2.0028	10.2	82.0	2.5(3H)	[16]
EtHC=N^•^	*c*-C_3_H_6_	220	2.0028	9.6	79.5	2.8(2H), 0.5(3H)	[20]
PhHC=N^•^	CCl_4_	270	2.0031	10.0	80.1	0.4(2H), 0.3(1H)	[17]
ArHC=N^•^ (**11**)	PhBu-*t*	300	2.0034	10.7	84.0	-	[21]
Me_2_C=N^•^	*c*-C_3_H_6_	223	2.0029	9.6	-	1.4(6H)	[19]
PhMeC=N	PhBu-*t*	308	2.0030	10.0	-	0.8(3H)	[23]
BnMeC=N^•^ (**9**)	PhBu*-t*	240	2.0033	9.8	-	1.5(3H), 1.1(2H)	[tw]
Ph_2_C=N^•^	CCl_4_	308	2.0033	10.0	-	0.4(8H)	[24]

^a^ Isotropic *g*-factors; isotropic hfs in Gauss; tw = this work.

The SOMO and isotropic spin density distribution computed for the biphen-2-yliminyl radical at the B3LYP/6-311+G(2d,p) level are illustrated in Figure 2b,c, respectively. These show rather clearly the orientation *in the nodal plane* of the C=N π-bond of lobes containing the upe and fully support the conclusions from the EPR data.

**Scheme 3:**
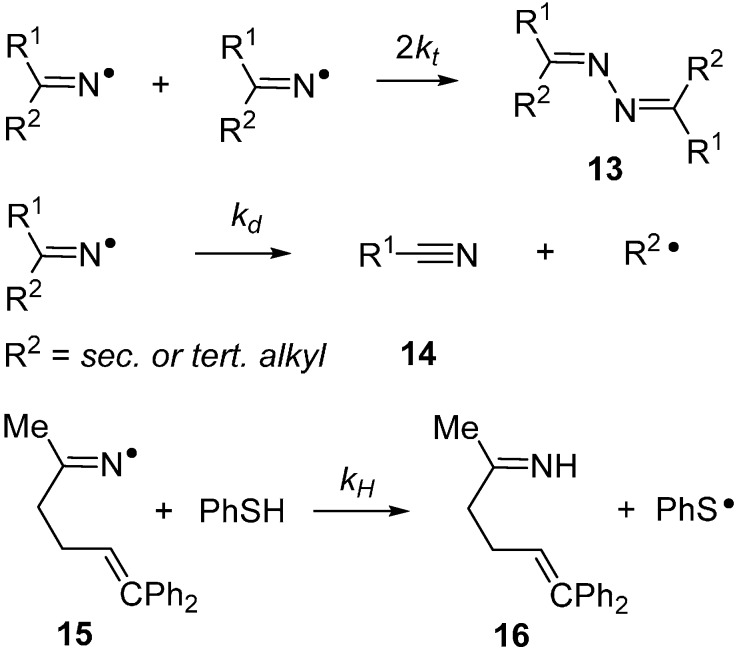
Combination, dissociation and H-atom abstraction reactions of iminyl radicals.

End product characterizations and EPR observations with iminyls [16,25] demonstrated that they terminate by bimolecular combination with production of bismethylenehydrazines **13** (Scheme 3) or by combination with alkyl radicals to yield imines. Ingold and co-workers established that these termination reactions are fast and diffusion controlled for small iminyls (2*k_t_* = 4 × 10^7^, 2 × 10^8^, and 4 × 10^9^ M^−1^·s^−1^ at 238 K for R^1^ and R^2^ i-Pr, Ph, and CF_3_, respectively) [18].

As expected, sterically shielded iminyls like *t*-Bu_2_C=N^•^ terminated much more slowly (2*k_t_* = 4 × 10^2^ M^−1^·s^−1^ at 238 K). Iminyl radical termination reactions and rates are, therefore, quite similar to those of *C*-centred analogues.

Iminyls also undergo dissociation by β-scission with production of nitriles **14** and release of *C*-centred radicals (Scheme 3). When R^1^ and R^2^ are aryl or *primary*-alkyl groups, this dissociation is slow at room temperature and does not compete with, for example, ring closure. However, at higher temperatures, and for R^1^ or R^2^ = *sec*- or *tert*-alkyl, β-scission is rapid. For example, if R^1^ = R^2^ = *t*-Bu then *k_d_* = 42 s^−1^ at 300 K [18]. Occasionally, iminyl radical dissociations have been put to use in preparations of organic nitriles [14,26]. Iminyl radicals are also known to abstract H-atoms from suitable substrates producing imines (**16**) and *C*-centred radicals. Kinetic studies are scarce but the diphenylhex-5-en-2-iminyl radical **15** abstracts H-atoms from thiophenol with a rate constant of 6 × 10^6^ M^−1^·s^−1^ at 298 K [27]. Comparing with *C*-centred analogues suggests that iminyls abstract H-atoms 10–20 times more slowly.

Iminyl radicals with alkenyl side chains ring close selectively in the *5-exo* mode with formation of pyrrolomethyl type radicals. This cyclisation forms the basis of several preparative procedures of pyrrole and dihydropyrrole containing heterocycles [26,28,29,30]. Some rate data has been determined by LFP [27] and steady state kinetic EPR methods [31], and key data is displayed in Table 2.

**Table 2:** Rate parameters for cyclisations of unsaturated iminyl radicals in solution ^a^.

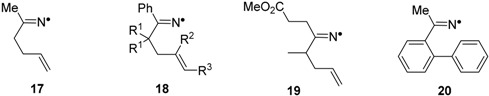			
**Radical; R^1^, R^2^, R^3^**	**mode**	***k_c_*/s**^−**1**^ **(300 K)**	***E_c_*/kcal·mol^−1^**
**17** ^b^	*5-exo*	10 × 10^3^	9.2
**18**; H, H, H	*5-exo*	8.8 × 10^3^	8.3
**18**; H, Me, H	*5-exo*	0.15 × 10^3^	10.7
**18**; H, H, Et	*5-exo*	60 × 10^3^	7.2
**18**; Me, H, H	*5-exo*	0.31 × 10^3^	10.3
**19**	*5-exo*	22 × 10^3^	7.8
**20** ^c^	*6-endo*	<5 × 10^3^	>9

^a^ Data from reference [31] except as indicated otherwise; all in hydrocarbon solution. Arrhenius *A*-factors assumed to be log(*A_c_*/s^−1^) = 10.0; ^b^ Data from references [31,32]; ^c^ Data from reference [33].

The rate constants for *5-exo*-cyclisations of the iminyls **17** and **18** (R^2^ = R^2^ = R^3^ = H) are smaller than that of the archetype *C*-centred hex-5-enyl radical by a factor of about 25. A Me substituent at the attacked end of the double bond reduced *k_c_* but an Et substituent at the terminus of the C=C double bond increased *k_c_* (Table 2). These trends are the same as observed with C-centred radical cyclisations. Surprisingly, the bismethyl substituted iminyl **18** (R^1^ = Me, R^2^ = R^3^ = H) displayed an inverse gem-dimethyl effect (Table 2). However *k_c_* for iminyl **19**, lacking the Ph substituent, but with a single Me in its pentenyl chain, showed the expected increase in *k_c_*. Thus, the inverse gem-dimethyl effect is specially related to the presence of the Ph substituent on the imine centre. DFT computations implied it was due to steric interaction between this Ph and the bis-Me groups in the alkenyl chain [31]. DFT computations of ring closure reactions of *C*-, *N*- and *O*-centred radicals with the high-quality quantum composite method Gaussian-4 [34] suggested that the stiffness and lack of flexibility associated with the C=N bond was responsible for the comparatively slow ring closure of iminyl radicals, rather than any effect from their higher electronegativity. In general, iminyls with aromatic acceptors such as **20** cyclised in *6-endo* mode with production of 6-member rings (see, however, Section 2.7). Kinetic data is sparse but implies that the rate constants for iminyl ring closures onto aromatics are also about an order of magnitude less than those of *C*-centred analogues [33].

### 2.3. Radical Based Transformations of Oxime Esters and Dioxime Oxalates

**Scheme 4:**
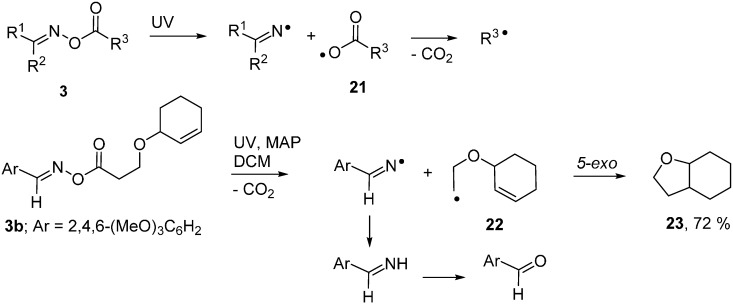
Photo-induced oxime ester transformations [22].

Oxime esters were the first oxime derivatives to be used for radical generation and they remain the most popular. They have been put to use in two ways; either as sources of *C*-centred radicals or for generating iminyl radicals. On homolysis of their weak N–O bonds, an iminyl radical is accompanied by an acyloxyl type radical **21** (Scheme 4). Most acyloxyls decarboxylate and release *C*-centred radicals very rapidly [35] making these precursors very effective sources of the latter. UV photolyses of appropriate **3**, with and without sensitisers, have proved effective for generating a broad range of *primary*, *secondary* and *tertiary* *C*-centred radicals as well as allylic types and even σ-radicals such as cyclopropyl and trifluoromethyl [21,22].

The good quality EPR spectra obtainable enabled the configurations and conformations of these radical types to be elucidated. The reactions they underwent with their parent oxime esters, or with other partners or co-reactants (see Figure 1b for an example), were also monitored. A recent time-resolved EPR spectroscopic investigation of the photo-cleavage of several oxime esters enabled effective spin-spin relaxation times *T*_2_* to be determined [36]. From the *T*_2_* dependences on monomer concentrations, rate data was deduced for addition reactions with acrylate monomers.

Oxime esters **3** proved to be clean and convenient sources of C-centred radicals that could be used in syntheses of various alicyclic and heterocyclic compounds [22]. Scheme 4 depicts one example in which photolysis of precursor **3a** released cyclohexenyloxyethyl radical **22** which underwent *5-exo*-ring closure and yielded octahydrobenzofuran **23** after H-atom transfer. The accompanying iminyl radical simply abstracted H-atoms with production of an imine that was hydrolysed to the easily separable aldehyde during work-up.

*O*-Acetyl oxime esters R^1^R^2^C=NOC(O)Me were established as highly effective sources of iminyl radicals for synthetic purposes because the partner MeC(O)O^•^ radicals were simply converted to volatile CO_2_ and CH_4_ [37,38,39]. Because symmetrical dioxime oxalates also cleanly yield only one iminyl radical, they too proved to be very successful in UV promoted preparative procedures [40,41]. Convenient photochemical routes, starting from these precursors, were described for pyrroles, dihydropyrroles, quinolines, phenanthridines and other aza-arenes with a range of functionality.

### 2.4. Oxime Esters and Photoredox Catalysis

Recently, in the interests of environmental protection and energy efficiency, research has expanded dramatically into finding procedures that require only visible light, plus catalytic quantities of some special promoter. For generating radicals, photoredox catalysts (PCs) of several types that require only visible light (sometimes UVA) have been developed. Heterogeneous PCs are mainly inorganic semiconductors, particularly titania (TiO_2_), which has found numerous applications in conjunction with allylic alkenes, alkyl amines, carboxylic acids and other compounds [42,43,44,45]. Development of the alternative homogeneous type PCs has flourished exceptionally well. The most widely used members of this class are complexes of Ru or Ir, particularly Ru(bpy)_3_^2+^ and *fac*-[Ir(ppy)_3_], that usually operate in conjunction with bromocarbonyl compounds and other organic halides [46,47,48,49,50]. Most PCs adsorb a photon from the incident light and are thereby raised to a long lived triplet state PC* that can act both as a reductant and/or an oxidant. In the presence of an acceptor molecule A, with a suitable redox potential, electron transfer from the PC* generates the radical anion A^−•^. These radical anions then convert to neutral radicals A^•^ by loss of an anion such as halide X^−^ or carboxylate. Alternatively, PC* may accept an electron from a suitable donor molecule D, thus creating the radical cation D^+•^ that then converts to a neutral radical by loss of a cation, usually H^+^.

**Scheme 5:**
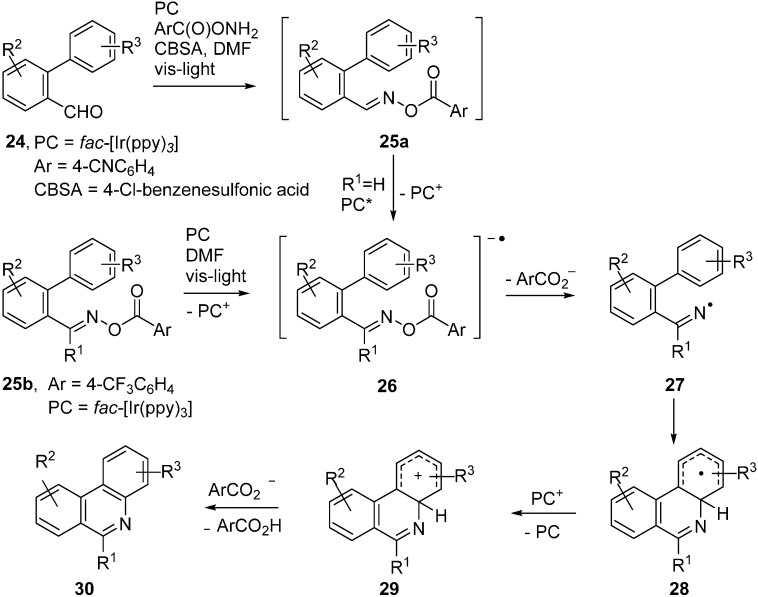
Reactions of designer oxime esters catalysed by *fac*-[Ir(ppy)_3_] [51,52].

Expressly designed oxime ester types were trialled recently to function as acceptors with *fac*-Ir(ppy)_3_ as PC [51,52]. Oxime esters **25a**,**b**, containing *O*-benzoyl moieties substituted with strong electron withdrawing groups such as 4-CN and 4-CF_3_, were shown to be effective in this role (Scheme 5).

Electrons were transferred to these oxime esters from the [*fac*-Ir(ppy)_3_]* triplet states so generating transient radical anions **26**. Loss of the substituted benzoate anions from **26** generated iminyl radicals **27**. Cyclisation onto the adjacent aryl rings afforded cyclohexadienyl type radicals **28b**. Oxidation to the corresponding cyclohexadienyl type cations **29** took place via SET to the previously produced PC^+^ ions. Proton loss then led to the product phenanthridines **30**. A wide range of the latter was obtainable in this way and, for **24** with Ar = 4-CNC_6_H_4_, the procedure could be carried out in one pot from the aldehydes without isolation of **25a** (Scheme 5). Analogous PC mediated routes to quinoline and pyridine derivatives were also demonstrated.

### 2.5. Photodissociation of Ketoxime Glyoxalates

**Scheme 6:**
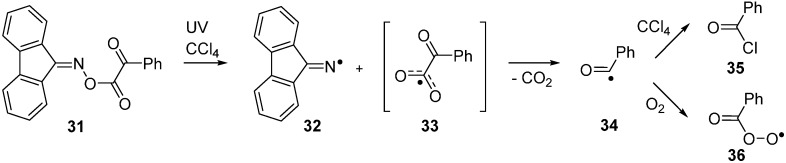
Photodissociation and subsequent reactions of an oxime glyoxalate [53].

Bucher and co-workers investigated the LFP induced dissociation of 9-fluorenone oxime phenylglyoxylate **31** in CCl_4_ solution by a variety of spectroscopic methods [53]. They used time resolved FTIR and time-resolved EPR to identify intermediate radicals. By these means, dissociation was shown to produce the expected iminyl radical **32** together with very short-lived benzoylcarbonyloxyl radical **33**. Rapid loss of CO_2_ from **33** yielded benzoyl radicals **34** and their subsequent chemistry dominated the system (Scheme 6).

Benzoyl radicals abstracted chlorine atoms from solvent to yield benzoyl chloride **35b** (or a bromine atom from CCl_3_Br to yield benzoyl bromide). In the presence of oxygen, coupling occurred with generation of peroxyls **36**. Preparative sequences based around oxime glyoxalates have not so far been established, but it seems clear they could be exploited as new clean sources of acyl or aroyl radicals as well as for iminyl radicals.

### 2.6. Carbamoyl Radicals from Oxime Oxalate Amides: Ring Closures to β- and γ-Lactams

**Scheme 7:**
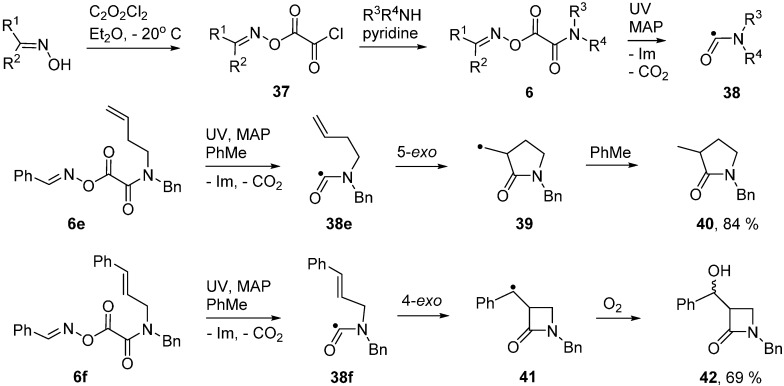
Oxime oxalate amides and ring closures of carbamoyl radicals [54,55].

Oxime oxalate amides **6** may be obtained in good yields from *O*-chlorooxalyl oximes **37** and amines (Scheme 7).

UV photolyses of solutions in PhBu-*t* delivered exclusive scissions of their weak N–O bonds. A number of iminyl and, after CO_2_ extrusion, carbamoyl (aminoacyl) radicals **38**, were observed by EPR spectroscopy [54,55]. Some representative EPR data for carbamoyls, including literature values, is collected in Table 3. The N–C bonds of amides have partial double bond character and, hence, high internal rotation barriers (20 ± 5 kcal·mol^−1^). High barriers are therefore expected in carbamoyl radicals. Experimental data is lacking, but a DFT computation [B3LYP/6-311+G(2d,p)] on radicals **38a** and **38b** provided an internal rotation barrier (about the N–C(O) bond) of 19.8 kcal·mol^−1^. It follows that carbamoyls will be capable of existing as *E* and *Z* isomers. Not surprisingly, only one isomer, presumably the *E*-isomer, was detected in each case. Carbamoyls such as **38b**, with *cis*-structures in which the NH hydrogen is *trans* to the orbital containing the upe, have been generated by H-abstraction from *N*-alkyl formamides [56]. The hfs from the NH hydrogens of these conformers are of much larger magnitude (see Table 3).

**Table 3:** EPR parameters of carbamoyl (aminoacyl) radicals in solution.

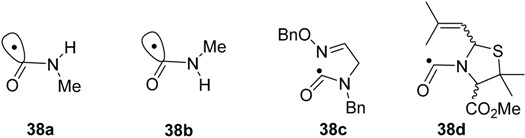						
**Radical**	**Solvent**	**T/K**	***g*-Factor**	***a*(N)**	***a*(Other)**	**Reference**
Me(H)NC^•^(O) (**38a**, *trans*)	PhMe	208	2.0015	24.0	0.9(NH), 0.9(3H)	[56]
Me(H)NC^•^(O) (**38b**, *cis*)	PhMe	208	2.0015	21.2	25.1(NH)	[56]
but-4-enyl(Bn)NC^•^(O) (**38e**)	PhBu-*t*	230	2.0017	21.7	0.8(1H)	[55]
but-2-enyl(Bn)NC^•^(O)	DTBP	360	2.0019	22.1		[57]
*n*-Bu(Bn)NC^•^(O)	DTBP	360	2.0019	21.9	0.9(4H)	[57]
**38c**	PhBu-*t*	220	2.0018	23.3		[58]
**38d**	PhBu-*t*	220	2.0018	21.0	1.6(1H)	[55]

**Figure 3:**
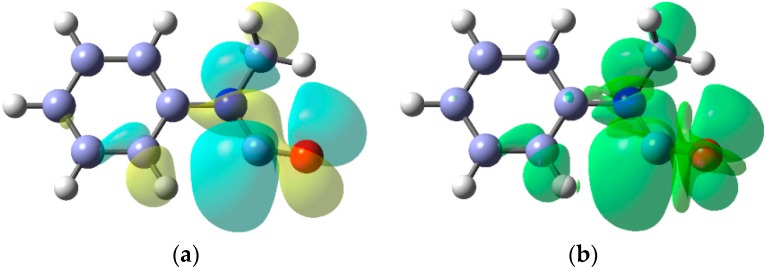
(**a**) DFT computed alpha SOMO of Ph(Me)NC^•^(O) radical; (**b**) DFT computed spin density distribution of Ph(Me)NC^•^(O) radical.

A noteworthy feature of carbamoyl radicals’ EPR spectra is that their *g*-factors are comparatively small and actually less than that of the free electron (*g* = 2.0023). Only a few other radicals share this characteristic so the *g*-factor is an aid in identification [59]. The comparatively large *a*(N) hfs indicate carbamoyls have σ-electronic structures and this was supported by DFT computations. Figure 3 shows the σ-lobe of the SOMO of the Ph(Me)NC^•^(O) radical, computed at the B3LYP/6-311+G(2d,p) level, *in the nodal plane* of the CO π-system. The spin density distribution (Figure 3b) mirrors this feature.

Carbamoyl radicals containing suitably situated acceptor groups readily underwent ring closure. For example, the *N*-but-4-enyl oxime oxalate amide **6e** furnished the carbamoyl radical **38e** and this cyclised efficiently in the normal *5-exo*-mode to afford pyrrolidin-2-one derivative **40** in good yield [55] (Scheme 7). Interestingly, carbamoyl radicals functionalised with allylic side chains also underwent ring closure in the rare *4-exo*-mode with eventual production of 4-member ring azetidin-2-ones. For example, precursor **6f** produced carbamoyl **38f** that, after *4-exo*-cyclisation to azetidinylalkyl radical **41**, yielded β-lactam **42**.

**Scheme 8:**
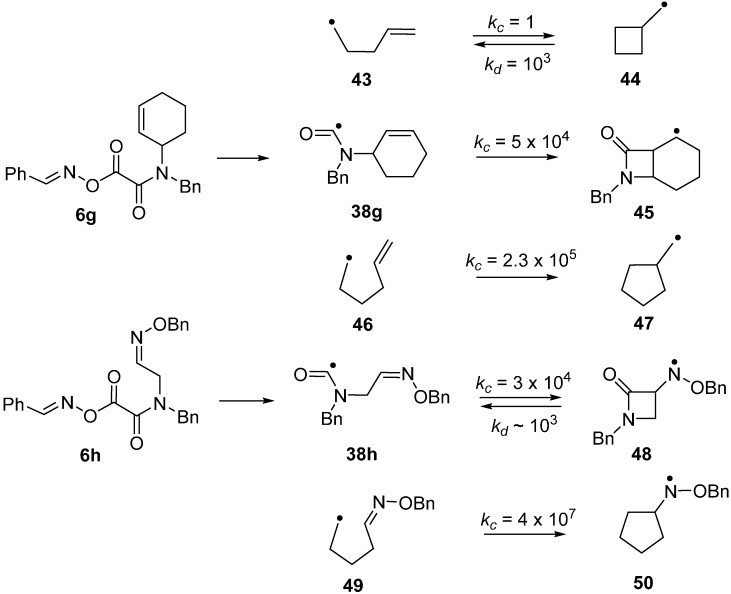
Kinetic data for ring closures of carbamoyl and model radicals at 300 K in solution. Rate constants *k*/s^−1^ [54,73,74,75,76].

The azetidinone ring system occurs as a key feature in the β-lactam family of antibiotics so preparative methods are of special interest. Radical closures to 4-member rings are normally slow in comparison to the reverse ring opening which is fast because of the strain in the 4-member rings [60,61,62]. Note the rate constants (*k_c_*) for 4-*exo*-closure of the but-3-enyl **43** and ring opening (*k_d_*) of the cyclobutylmethyl radical **44** in Scheme 8. The 4-member azetidinone ring system has proved to be exceptional because β-lactams have been prepared by cyclisations of carbamoyl radicals [63,64,65], of amidoalkyl radicals [66,67,68,69,70] and of amidyl radicals [71]. This topic has been reviewed in the context of homolytic ring closures in general [72]. EPR spectra obtained from UV irradiations of oxime oxalate amides made possible kinetic studies of several carbamoyl radical cyclisations [58,73]. Carbamoyl **38g** was obtained from photodissociation of precursor **6g** and the rate constant for its *4-exo*-cyclisation onto a C=C bond to yield bicyclic radical **45** was determined to be four orders of magnitude greater than that of archetype pent-4-enyl radical **43** (Scheme 8). The rate constant for 4*-exo*-cyclisation of carbamoyl **38h** on to a C=N bond to produce aminyl radical **48** was of the same order of magnitude. Note that the rate constant for opening of the azetidinone ring of **48** was not much different from that of model **44** but was sufficiently smaller than *k_c_* (for **38b**) so that ring closed products could be isolated. As expected, all the *4-exo* rate constants were smaller than the archetype *k_c_* for *5-exo* cyclisation of hex-5-enyl **46** [74,75] and analogue **49** [76] (Scheme 8). It is worth mentioning that *4-exo*-ring closures of cyclic carbamoyl radicals containing thiazolidine rings **38d**, to directly give bicyclic penicillanic structures, were not achieved [73].

### 2.7. Dissociation of Oxime Carbonates and Generation of Alkoxycarbonyloxyl Radicals

**Scheme 9:**
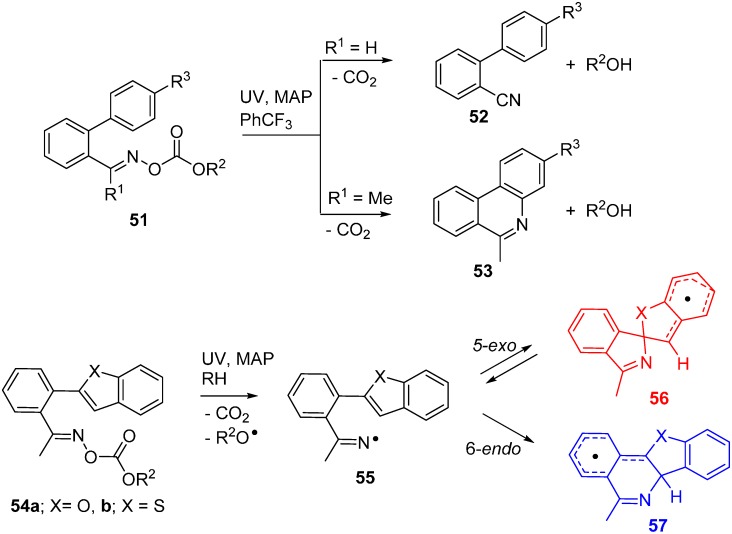
Reactions of iminyl radicals derived from oxime carbonates [33,78].

Oxime carbonates **51**, like most of the fore-going oxime esters, have a bi-modal character and can operate either as sources of iminyl radicals or for production of the little known alkoxycarbonyloxyl radicals, depending on the pattern of functionality [77]. For generation of iminyls, oxime carbonates with *O*-ethoxycarbonyl **51** (R^2^ = Et) or *O*-phenoxycarbonyl **51** (R^2^ = Ph) substitution were easily prepared by condensation of oximes with the corresponding chloroformates. Photolyses of the aldoxime derived precursors **51** (R^1^ = H) in benzotrichloride solvent afforded nitrile products **52**. However, good yields of phenanthridines **53** with a range of functionality were obtained from **51** (R^1^ = Me) (Scheme 9). The by-products, ethanol or phenol (R^2^OH), were easily separated [23,78].

An intriguing dichotomy of behaviour was observed during transformations of oxime carbonates functionalised with benzofuran **54a** and benzothiophene **54b** groups. In biphen-2-yliminyl radicals (e.g., **20**), the SOMO on the N-atom is well placed for orbital overlap at either the *ipso*-C-atom (*5-exo*-ring closure) or the *ortho*-C-atom (*6-endo*-ring closure); see Figure 2b,c (Section 2.2). In analogous fashion, iminyls **55a** and **b** were capable of undergoing *either* *5-exo*-ring closure to *spiro*-radical **56a**,**b** or *6-endo*-ring closure to **57a**,**b**. In preparative photolyses with **54a** and **54b** at ambient temperature (~330 K) exclusively, the benzofuro[3,2-*c*]isoquinoline and benzo[4,5]thieno[3,2-*c*]isoquinoline products derived from the *endo*-radicals **57a** and **57b** were isolated in good yields [78]. Curiously, EPR spectra taken during photolyses of **54a** and **54b** in solution at 230 K showed solely the *spiro*-radicals **56a** and **56b** [33]. The structures and energies of the iminyl and cyclised radicals were computed by DFT at the B3LYP/6-311+G(2d,p) level of theory with the Gaussian 09 package [79].

**Figure 4:**
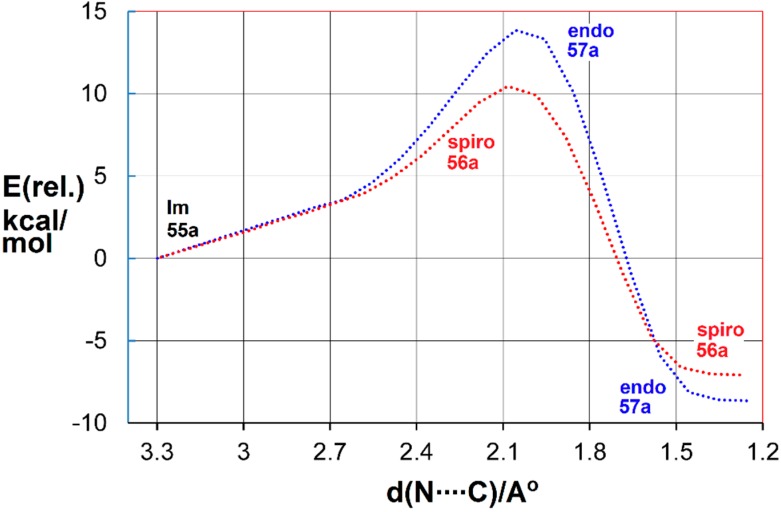
DFT computed reaction coordinates for *spiro* (**red**) and *endo* (**blue**) ring closure of benzofuro-iminyl radical **55a**.

The transition states for *spiro* and *endo* ring closure were also located. The intrinsic reaction coordinate scans are illustrated in Figure 4 for the benzofuro system **55a**.

The DFT computations showed *spiro* ring closure to be exoenthalpic (*∆H*_298_ = −6.9 kcal·mol^−1^) but the *endo* mode was thermodynamically more favourable (*∆H*_298_ = −8.1 kcal·mol^−1^). However, the computed enthalpy of activation for *spiro*-closure (*∆E^‡^*_298_ = 9.3 kcal·mol^−1^) was significantly lower than that for *endo*-closure (∆*E^‡^*_298_ = 13.1 kcal·mol^−1^) (see Figure 4). Similar trends were computed for the benzothieno system from radical **55b**. These DFT energies supported the conclusion that, at the low temperature of the EPR experiments, *kinetic* control led to *spiro* radicals **56a**,**b**. However, at the temperature of the preparative experiments (~100 K higher), the *spiro*-cyclisations were reversible but the *endo* were not. Consequently, thermodynamic control steered the processes towards accrual of the *endo* products. The alternative possibility that the *spiro*-radicals **56a**,**b** rearranged to the *endo* radicals **57a**,**b** by 1,2-shifts via tetracyclic structures could be discounted because DFT computations indicated much higher energy requirements.

In the past, a few alkoxycarbonyloxyl radicals [ROC(O)O^•^] had been generated from hazardous dialkyl peroxydicarbonate precursors (**58**) and studied by EPR spectroscopy [80] and LFP [81]. By means of suitably functionalised oxime carbonates, a much wider range of these radicals became accessible and their chemistry could be explored in greater detail.

**Scheme 10:**
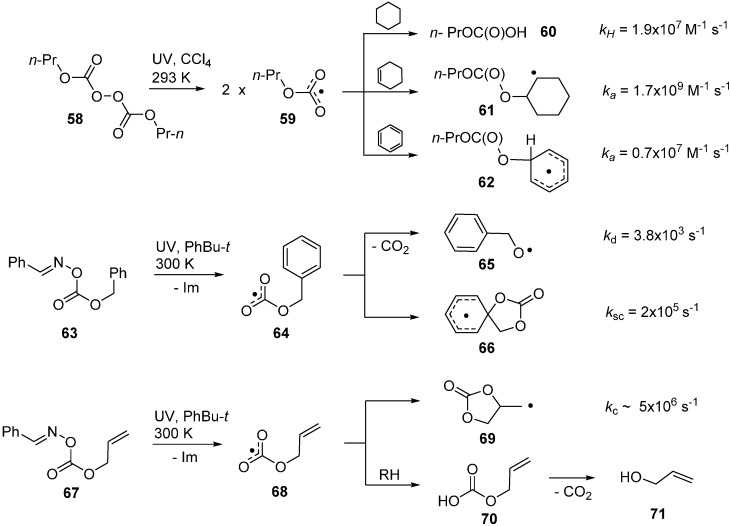
Reaction channels and rate constants for alkoxycarbonyloxyl radicals [23,81,83].

Remarkably, alkoxycarbonyloxyl radicals dissociate to release CO_2_ much more slowly than acyloxyl radicals (RCO_2_^•^). Thus, they have sufficient lifetimes to participate in a range of abstraction, addition and cyclisation processes. They are not directly detectable by EPR spectroscopy [80] due to fast relaxation times but their broad absorptions centred at around 640 nm in the UV-visible spectrum have been observed in the LFP studies [81]. They are very reactive and, for example, propyloxycarbonyloxyls **59** abstract H-atoms from secondary CH_2_ sites to produce esters of carbonic acid **60** with a rate constant of 1.9 × 10^7^ M^−1^·s^−1^. They add to unactivated alkenes nearly a factor of 100 faster and also add to aromatics to produce cyclohexadienyl type radicals **62** with ease (see Scheme 10). In accord with this, the methoxycarbonyloxyl radical MeOC(O)O^•^ was also observed to add to the alkenic sites of lipid components more rapidly than it abstracted allylic type H-atoms [82]. Furthermore, EPR spectroscopic data showed it dissociated to CO_2_ and MeO^•^ radicals with a rate constant of about 2 × 10^3^ s^−1^ at 300 K. Oxime carbonate **63** released benzyloxycarbonyloxyl radical **64** and surprisingly this (and derivatives) underwent exclusively *spiro*-cyclisation to produce *spiro*-cyclohexadienyl radical **66** [23,83]. From steady state kinetic EPR concentration measurements, the rate constant for CO_2_ loss from **64** was determined to be 3.4 × 10^3^ s^−1^ at 300 K. The similarity in the *k_d_* values for MeOC(O)O^•^ and BnOC(O)O^•^ verified that alkoxycarbonyloxyls lose CO_2_ about seven orders of magnitude more slowly than acyloxyl radicals.

The rate constant for *spiro*-cyclisation was measured to be 2 × 10^5^ s^−1^ and this is about an order of magnitude greater than for *spiro*-cyclisation of C-centred analogues [84,85,86]. Similarly, oxime carbonates with allylic side-chains, e.g., **67** released the corresponding allyloxycarbonyloxyl radicals **68** on photolysis. The latter ring closed in the *5-exo*-mode to produce 1,3-dioxolan-2-on-4-ylmethyl radicals **69** very rapidly. The cyclisation rate constant (*k_c_* = 5 × 10^6^ s^−1^ at 300 K) was obtained from kinetic EPR measurements. Scheme 10 illustrates that the cyclisation rates of alkoxycarbonyloxyl radicals onto aromatic and alkenic acceptors are significantly faster than analogous rates of C-centred radicals. In this respect, they resemble alkoxyl radicals, such as pent-4-enyloxyl, that are also known to ring close much more rapidly than *C*-centred analogues [87,88]. Factors influencing the rapidity of *5-exo*-cyclisations of *C*-, *N*- and *O*-centred radicals have been reviewed [34].

Products containing the 1,3-dioxolan-2-one unit (cyclic carbonates) were isolated from cyclisations of species such as **69**; though in meagre yields. H-atom abstractions from solvents or substrates by alkoxycarbonyloxyl radicals were also rapid so that alkylcarbonate esters such as **70** formed very easily. These were mono-esters of carbonic acid and as such were known to be unstable and extrude CO_2_ [89,90]. The resulting alcohols e.g., **71** were usually the major products isolated from alkoxycarbonyloxyl reactions. A synthetic protocol to trap and isolate products containing the cyclic carbonate structural unit has yet to be devised.

### 2.8. Oxime Carbamates: Precursors for Iminyl and Aminyl Radicals

Previous interest in oxime carbamates **71** (*O*-carbamoyl oximes) centred on their biological activities [91,92] and their known inhibition of several enzymes [93,94]. Designer compounds of this class have recently been adapted for release of specific radicals [95]. UV irradiation leads again to selective scission of their N–O bonds with formation of iminyls together with the rare and exotic carbamoyloxyl radicals **72**. DFT computations predicted that *N,N*-di-substituted carbamoyloxyls would dissociate very rapidly to CO_2_ and di-substituted aminyl radicals **73**. However, the computations implied that *N*-mono-substituted carbamoyloxyls (**72**, R^1^ or R^2^ = H) might have sufficient lifetime to be trapped at low temperatures. Study of UV photolyses of a set of oxime carbamates in solution by EPR spectroscopy revealed that for *N,N*-di-substitution *only* Im and aminyl radicals **73** were produced in the accessible temperature range. However, with the mono-substituted *N*-allyl-precursor **71** (R^1^ = H, R^2^ = allyl), the EPR spectrum of the oxazolidinylmethyl radical **76**, from ring closure of the corresponding carbamoyloxyl radical **75**, was discerned at about 150 K. So, this *N*-mono-substituted example did have sufficient structural integrity to undergo *5-exo*-cyclisation; in confirmation of the theoretical prediction.

At room temperature and above, decarboxylation is rapid so both *N*-mono- and *N,N*-di-substituted oxime carbamates provide much needed benign and serviceable alternatives for aminyl radical generation. The EPR parameters of a representative set of aminyl radicals obtained mainly in this way are listed in Table 4. It should be noted that only a very few *N*-monoalkylaminyl radicals have been detected in solution; though monoarylaminyls do provide good isotropic spectra in which the upe is delocalised into the ring [96].

**Figure 5:**
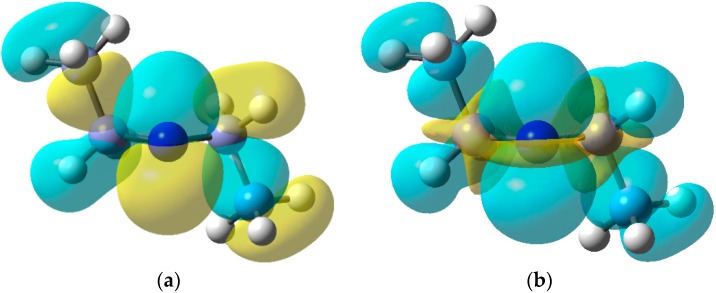
(**a**) DFT computed SOMO for diethylaminyl radicals; (**b**) Spin density distribution.

**Scheme 11:**
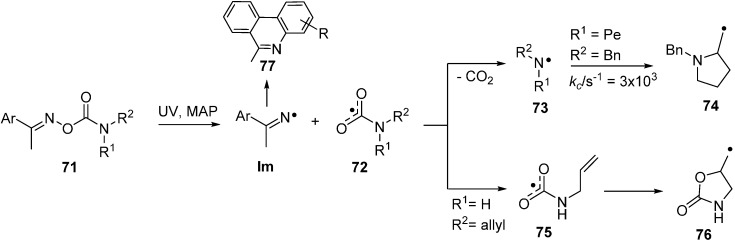
Photochemical reactions of oxime carbamates [95].

The EPR data indicate that dialkylaminyl radicals are bent; the magnitudes of the *a*(N) values are consistent with π-type electronic configurations. The DFT computed SOMO and spin density [B3LYP/6-311+G(2d,p)] for the Et_2_N^•^ radical in Figure 5 clearly show the π-type orbital associated with the N-atom and the considerable spin density distributed to the Et groups. In this respect, aminyl radicals resemble the familiar *C*-centred alkyl radicals although, as the cyclisation rate constant in Scheme 11 illustrates, they generally react more slowly.

**Table 4:** Isotropic EPR parameters for selected dialkylaminyl radicals R_2_N^• a^.

Radical	T/K	*g*-Factor	*a*(N)/G	*a*(H^β^)/G	*a*(H^β^)/G	Reference
Me_2_N^•^	183 ^b^	2.0044	14.8	27.4 (6H)		[97,98]
Et_2_N^•^	210	2.0047	14.4	35.7 (2H)	35.7(2H)	[95]
Bn_2_N^•^	220	2.0046	14.3	37.1 (2H)	37.1 (2H)	[95]
allyl_2_N^•^	210	2.0048	14.6	36.0 (2H)	36.0 (2H)	[95]
BnN^•^Pe	230	2.0048	14.2	36.9 (2H)	35.4 (2H)	[95]

^a^ In PhBu-*t* solution unless otherwise specified; ^b^ In cyclopropane solution.

The viability of the alternative mode of oxime carbamate usage, as precursors of iminyl radicals (**Im**), was effectively demonstrated with diethyl substituted precursors **71** (R^1^ = R^2^ = Et). Good yields of phenanthridines were obtained when the Ar group was a biphenyl moiety. Photolysis of precursor **71** with a pent-4-enyl (Pe) chain released aminyl radical **73** (R^2^ = Bn, R^1^ = Pe) that was characterised by EPR spectroscopy (Table 4). Cyclisation took place above about 250 K and ring closure kinetic parameters were derived for this *N*-centred species (Scheme 11).

## 3. Oxime Ethers in Radical-Mediated Reactions

### 3.1. Homolytic Reactions of O-alkyl and O-aryl Oxime Ethers

**Scheme 12:**
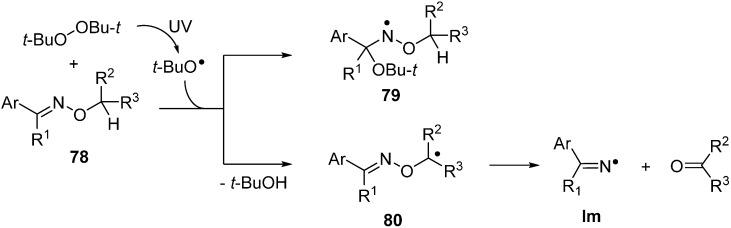
Radical addition to oxime ethers and radical induced dissociations [99].

The N–O bonds of most oxime ethers **78** do not readily undergo homolysis on irradiation with UVA or UVB and only low conversions to ketones and/or nitriles could be achieved even after prolonged photolyses [99]. Radicals of many types add to the C=N bonds of oxime ethers particularly rapidly [100]. Not surprisingly, therefore, when *t*-BuO^•^ radicals are generated in the presence of an *O*-alkyl, oxime ether **78** addition takes place with production of oxyaminyl radicals **79** (Scheme 12).

However, if the oxime ether contains an O–CH or O–CH_2_ group, then H-atom abstraction competes with addition such that *C*-centred radicals **80** are also formed. The latter readily undergo β-scission to release an iminyl radical together with an aldehyde or ketone (Scheme 12) [99]. The importance of abstraction relative to addition depends on the substitution pattern and temperature and consequently this is seldom a clean, selective mode of radical generation from oxime ethers. Photoredox catalytic systems for putting oxime ethers to work have now been developed (see below) but recent attention has focused mainly on thermolytic methods.

### 3.2. Conventional and Microwave Mediated Thermolyses of Oxime Ethers

Conventional thermal dissociations of oxime ethers R^1^R^2^C=NOBn can be brought about by heating them in hydrocarbon solvents at *T* > ~150 °C. However, products from scission of the N–O bond (BnOH and R^1^R^2^C=NH) together with products from O–C bond breaking (R^1^R^2^C=NOH and PhCH_3_) were obtained; so, these substrate types are not suitable as clean radical sources [101]. It was found, however, that *O*-phenyl ketoxime ethers R^1^R^2^C=N–OPh (R^1^, R^2^ = alkyl or aryl) undergo selective N–O homolysis upon heating in hydrocarbon solvents at moderate temperatures (*T* ~ 90 °C) to yield iminyl and phenoxyl radicals [6]. The substantial resonance stabilisation of the phenoxyl radical predisposes the homolysis in favour of N–O scission. In principle, this constituted a new and promising route to iminyl radicals because the phenoxyl radicals usually ended up as the acidic, and therefore easily separable, PhOH.

**Scheme 13:**
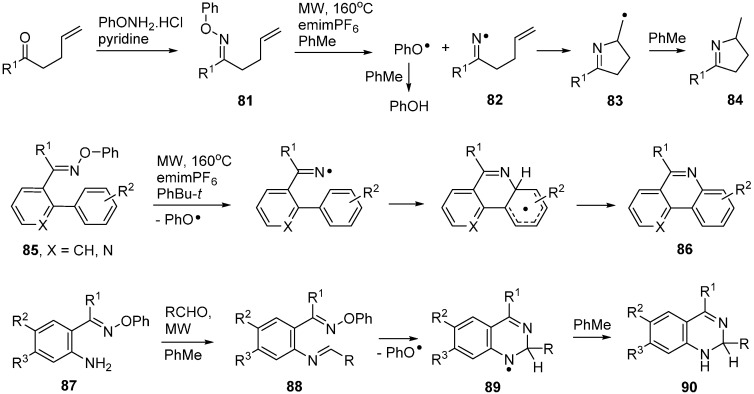
MW assisted preparations of dihydropyrroles, aza-arenes and quinazolines from *O*-phenyl oxime ethers [30,103].

Thermal methods are generally advantageous for preparative work because of their simplicity and ease of scale-up. In practice, however, conventional thermolyses of *O*-phenyl oxime ethers required long reaction times with consequent poor selectivity and yields. Fortunately, it was discovered that microwave heating (MW) was very advantageous for cleanly generating iminyl radicals from a large range of *O*-phenyl oxime ethers [29,30]. The optimum conditions for oxime ethers with alkene acceptors **81** involved MW irradiation at 160 °C for 15–30 min in an H-donor solvent such as toluene. The ionic liquid 1-ethyl-3-methylimidazolium hexafluorophosphate (emimPF_6_) was added to improve the microwave absorbance level of the medium. Intermediate iminyl radicals such as **82** underwent *5-exo* cyclisation to radicals **83** and these abstracted H-atoms from the solvent to afford good yields of 3,4-dihydropyrroles **84** (Scheme 13).

*O*-Phenyl oxime ethers containing appropriately placed aromatic acceptors **85** also dissociated cleanly under MW radiation. The final step required an oxidation in this case so PhBu-*t* proved to be a better solvent enabling aza-arenes of type **86** and others to be isolated in good yields. As a further elaboration of the process, 2-aminoarylalkanone *O*-phenyl oxime precursors **87** were prepared. Mixtures of these, with an equivalent of an aldehyde, on MW irradiation in toluene with emimPF_6_ as additive, initially yielded imines **88**. These were not isolated but dissociated, released iminyl radicals that cyclised exclusively in *6-endo* mode to produce aminyl radicals **89**. The latter were reduced to dihydroquinazolines **90** under the reaction conditions (Scheme 13) [102,103]. With ZnCl_2_ as additive to promote condensation the MW reaction proceeded in one pot to afford directly the oxidised quinazolines.

**Scheme 14:**
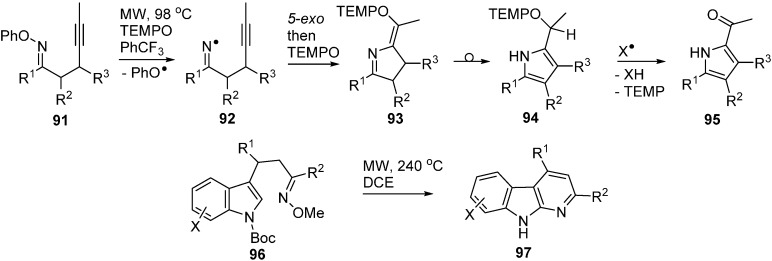
Cyclisations of *O*-phenyl and *O*-methyl oxime ethers [104,105].

A recent article described microwave promoted reactions of *O*-phenyl oxime ethers **91** with alkynyl side chains that afforded iminyls **92** as intermediates [104]. In the presence of excess TEMPO, these iminyls ring closed and then coupled with the TEMPO with production of dihydropyrrole intermediates **93** (Scheme 14). These rearranged to pyrrole structures **94** that spontaneously underwent H-atom transfer and fragmentation with production of 2-acylpyrroles **95**. The *O*-phenyl oximes **91** were easily obtained from ketones so the whole process provided ready access to a good range of functionalised pyrroles.

It is worth mentioning the report that indolyl-alkenyl *O*-methyl oxime ethers **96** were converted to pyridoindoles **97** (α-carbolines) on MW heating to 240 °C (Scheme 14) [105]. Superficially, the reaction resembles that of the *O*-phenyl oxime ethers but in this case the mechanism was believed to involve electrocyclisation.

### 3.3. Oxime Ethers and Photoredox Catalysis

**Scheme 15:**
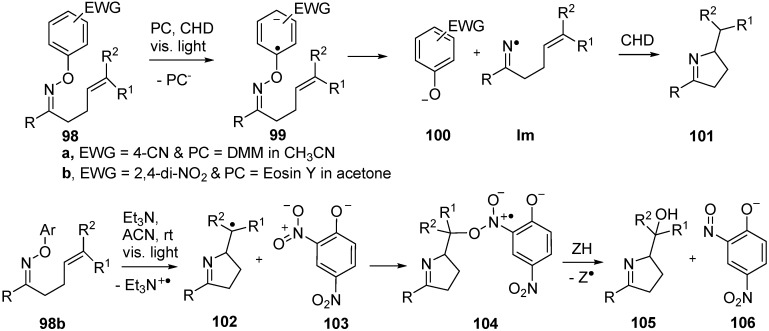
Photoredox catalyzed reactions of *O*-aryl oxime ethers [107,108].

*O*-Aryl oxime ethers containing electron withdrawing groups (EWG) in their aryl rings released iminyl radicals on irradiation with UVA or visible light when photoredox catalysts (PC) were employed. For example, oxime ether **98a** containing 4-CN (or 2,4-di-NO_2_ or 4-CF_3_) aryl substitution with a catalytic amount of 1,5-dimethoxynaphthalene (DMN) on irradiation with UV light (λ > 320 nm) in 1,4-cyclohexadiene (CHD) afforded good yields of dihydropyrroles **101** (Scheme 15) [28]. Electron transfer from the excited state of the PC generated the oxime ether radical anion **99** and this dissociated to give the arene-oxide **100a** together with the iminyl radical. The latter ring closed and the product **101** was formed by H-atom abstraction from the CHD H-donor present in excess.

More recently, it was shown that the reduction potentials of oxime ethers with *O*-2,4-dinitroaryl substitution **98b** were sufficiently low for the dye eosin Y to be used as the PC and then light of visible wavelength only was needed [106]. An additional interesting finding was that with **98b** Et_3_N could be employed, in place of eosin Y. Irradiation with visible light in CH_3_CN then led to isolation of imino-alcohols **105**. The proposed mechanism involved fast formation of an electron donor–acceptor complex between Et_3_N and the electron-poor ring of **98b**. Excitation with visible light then generated the radical anion analogous to **98** that fragmented to give stable phenoxide **103** and, after *5-exo*-cyclisation, pyrrolidinylmethyl radical **102**. Oxygenation took place by attack of radical **102** onto the NO_2_ group of **103** leading to intermediate **104**. Homolysis of the N–O bond of **104** gave nitroso-phenoxide **106** and an *O*-centred radical that rapidly abstracted hydrogen to furnish the product imino-alcohol **105** (Scheme 15) [107]. The scope of the process was found to be wide affording iminoalcohols in good to high yields from alkenes with a range of substituents and including bicyclic products.

In another interesting investigation, the *O*-methyl oxime ethers derived from 1,1′-biphenyl-2-carbaldehydes were shown to yield phenanthridine derivatives on treatment with visible light and catalytic 9,10-dicyanoanthracene [107]. However, the mechanism of this system was believed to involve photo-electron transfer to the PC with formation and cyclisation of radical cation intermediates.

## 4. Conclusions

It is clear that an appropriate oxime derivative can be found to provide a benign route, free of toxic metals, unstable peroxides or hazardous azo-compounds, to almost any radical centred on a first row element. These precursors have been exploited for uncomplicated production of known and exotic transient species, enabling the structures and reaction selectivity to be examined; particularly by EPR spectroscopy supported by DFT computations. In addition, they offer platforms for study of the kinetics of ring closures of *C*-, *N*- and *O*-centred radicals and for kinetic study of decarboxylations of several short-lived *O*-centred species. These compound types also deliver novel and convenient preparative procedures for aza-heterocycles containing both 5-member ring pyrrole type and 6-member ring pyridine structural units. Oxime ethers with *O*-aryl functionality proved particularly suitable for use with the convenient and innocuous MW technology [108]. There remains ample scope for development of synthetic protocols employing the acyl radicals from ketoxime glyoxalates and the aminyl radicals generated from oxime carbamates. Comparatively few O-containing heterocycles have been made from carbonyl oxime precursors so there is opportunity for developments in that area. Photoredox catalytic methods have been developed for specific oxime ester and oxime ether types. It seems certain that additional photoredox catalysts suitable for this purpose will be developed and applied to a wider range of oxime derivatives.
